# Comparative Analysis of Gut Microbiota between Captive and Wild Long-Tailed Gorals for Ex Situ Conservation

**DOI:** 10.3390/microorganisms12071419

**Published:** 2024-07-12

**Authors:** Chang-Eon Park, Young-Jae Jo, Da-Ryung Jung, Hee-Cheon Park, Jae-Ho Shin

**Affiliations:** 1 Department of Applied Biosciences, Kyungpook National University, Daegu 41566, Republic of Korea; aeonrapt@knu.ac.kr (C.-E.P.); dudwo7573@knu.ac.kr (Y.-J.J.); amugae1210@knu.ac.kr (D.-R.J.); 2 Institute of Ornithology, Ex Situ Conservation Institution Designated by the Ministry of Environment, Gumi 39105, Republic of Korea; cranesave@naver.com; 3 NGS Core Facility, Kyungpook National University, Daegu 41566, Republic of Korea

**Keywords:** conservation biology, ex situ conservation, long-tailed goral, gut microbiome

## Abstract

The long-tailed goral is close to extinction, and ex situ conservation is essential to prevent this phenomenon. Studies on the gut microbiome of the long-tailed goral are important for understanding the ecology of this species. We amplified DNA from the 16S rRNA regions and compared the microbiomes of wild long-tailed gorals and two types of captive long-tailed gorals. Our findings revealed that the gut microbiome diversity of wild long-tailed gorals is greatly reduced when they are reared in captivity. A comparison of the two types of captive long-tailed gorals confirmed that animals with a more diverse diet exhibit greater gut microbiome diversity. Redundancy analysis confirmed that wild long-tailed gorals are distributed throughout the highlands, midlands, and lowlands. For the first time, it was revealed that the long-tailed goral are divided into three groups depending on the height of their habitat, and that the gut bacterial community changes significantly when long-tailed gorals are raised through ex situ conservation. This provides for the first time a perspective on the diversity of food plants associated with mountain height that will be available to long-tailed goral in the future.

## 1. Introduction

According to the Korean Ministry of Environment, ~2000 long-tailed gorals inhabit Korea. However, to prevent the spread of African swine fever in long-tailed gorals, structures were installed in recent years to prevent the movement of all animals from north to south of Korea, leaving long-tailed gorals geographically isolated. In addition to these unfavorable conditions, the extremely heavy snow of last winter caused the deaths of approximately 1022 long-tailed gorals as they became further isolated. As a result, the long-tailed goral population declined rapidly.

Conservation activities are essential in preparing for population decline due to sudden environmental disasters. Ex situ conservation [1,2,3,4,5] refers to conservation in places other than the original habitat. The most common example of ex situ conservation is captivity in zoos [6,7,8,9,10]. Ex situ conservation, which is performed to prevent situations in which sudden events, such as natural disasters, lead to complete extinction due to a rapid decline in population, is important for preventing the extinction of a specific species.

In Korea, two representative organizations perform ex situ conservation: the National Park Service’s Northern Conservation Center and the Long-tailed Goral & Musk Deer Center. The two sites conserve the long-tailed goral in different ways. Both sites conserve and restore long-tailed gorals that are morphologically healthy. The National Park Service’s Northern Conservation Center mainly provides mulberry leaves, whereas the Long-tailed Goral & Musk Deer Center provides hay, mineral blocks, and leaves collected from the wild to the long-tailed goral.

It has not been revealed how much the conservation of Korean long-tailed goral differs between these two places depending on the feeding method, or how different it is from the wild. These differences were not detected in the past. However, the emergence of next-generation sequencing [11,12,13,14,15] has enabled analysis of the gut microbiome [16,17,18,19,20] to compare microecological differences.

If the ecology of the gut microbes of the long-tailed goral is microecology, the way the long-tailed goral interacts with its environment (wild or in captivity) can be viewed as macroecology. Microecology and macroecology are organic to each other [21,22]. In various animals, including humans, microecology is affected by changes in macroecology [23,24].

Long-tailed gorals form families or groups in the wild. Males build larger home ranges than females, giving up their home ranges to females and cubs where they can obtain food with less movement. They constantly move to avoid their natural enemies and therefore exercise sufficiently. Long-tailed gorals subsist on leaves, moss, rock minerals, soil, etc. In contrast, when raised under ex situ conservation, the long-tailed goral has no fear of natural enemies, has reduced movement in limited spaces, and subsists on limited food provided by humans.

The gut microbiome changes when the amount of exercise decreases [25,26,27] and when the food source changes [28,29,30,31,32]. On the basis of these facts, when conducting ex situ conservation from a conservation biology perspective, we must not only consider the macroecology of long-tailed gorals but also pay attention to their microecology and consider what changes occur in their microecology due to changes in their macroecology.

Outwardly, the long-tailed goral is doing well and may not appear to have any particular problems when reintroduced into the wild [33]. However, there are no scientific indicators as to how they are bred to differ from the wild through ex situ conservation and how close to the wild they are bred. The correct order would be to reintroduce long-tailed gorals after their breeding conditions have been verified using scientific and visual indicators. Because the gut microbiome of long-tailed gorals changes as they transition from the wild to captivity, we compared the gut microbiomes of long-tailed gorals raised under different conditions at two sites. We hypothesized that under similar exercise conditions, the gut microbiome will change depending on the diversity of food sources. Our findings indicate that studying the gut microbiome of long-tailed gorals will be useful for their conservation and reintroduction. Our study did not draw blood, measure body weight, measure feeding, control feeding, or measure exercise, and did not study the fungal microbiome. We clearly state that there are limitations in presenting the results by only conducting bacterial microbiota studies.

Our study is important because, to date, it has not been revealed how the bacterial microbiota of long-tailed gorals changes during captivity and to what extent their microbiota is modified at origin. The purpose of our study is to reveal for the first time how the bacterial microbiota of long-tailed gorals is modified during captivity, and from the results, to provide guidance on how to provide food plants for the conservation of long-tailed gorals.

## 2. Materials and Methods

### 2.1. Fecal Sample Collection

Feces were collected from wild long-tailed gorals at Seoraksan National Park. Feces from captive long-tailed gorals were collected from the National Park Service’s Northern Conservation Center and the Long-tailed Goral & Musk Deer Center (Table 1). Only fresh fecal samples that were not contaminated with soil were selected, collected using sterile nitrile gloves, and placed in sterile Ziploc bags. The sterile nitrile gloves were replaced after each collection. The samples placed in sterile Ziploc bags were immediately placed in a cold insulated bag, transported safely to the laboratory, and stored in a deep freezer at −80 °C until DNA extraction.

### 2.2. Bioinformatics Analysis

Fecal DNA was extracted from 250 mg of homogenized fecal solution using a QIAamp PowerFecal Pro DNA Kit (Qiagen, Hilden, Germany) according to the manufacturer’s protocol [34,35,36]. Total DNA was used as a template to amplify the 16S rRNA region of nuclear ribosomal DNA. The V4–V5 region of the 16S rRNA gene was amplified using the 515F–926R primer pair [37,38,39,40,41]. Deionized–distilled water was used as a negative control, and DNA from the fungal strain *Bacillus licheniformis* KACC 10476 was used as a positive control. Then, barcoded sequences were added to construct a sequencing library.

The PCR reaction for the sequencing library was performed in a 25-μL reaction, containing 12.5 μL of 2X EmeraldAmp PCR master Mix (Takara Biotechnology Co., Ltd., Shiga, Japan) [42], 0.1 μM (final concentration) of each primer, and 1 ng of template. PCR was performed using the following temperature program: 5 min of predenaturation at 95 °C, followed by seven cycles of denaturation (95 °C for 30 s), annealing (55 °C for 30 s), and extension (72 °C for 30 s); 25 cycles of denaturation (95 °C for 30 s), annealing, and extension (72 °C for 1 min); and final extension at 72 °C for 5 min. The prepared libraries were diluted to the same molecular weight, transferred to an Eppendorf tube, and purified using AMPure XP beads (Beckman Coulter, Brea, CA, USA) [43,44,45,46] to complete the final library.

The final library was diluted to 8 pM, and emulsion PCR was performed using ion sphere particles [47,48,49,50,51] with the Ion OneTouch 2 System (Thermo Fisher Scientific, Waltham, MA, USA) [52,53,54,55]. This was followed by enrichment of template-positive ion sphere particles using Dynabeads MyOne Streptavidin C1 beads (Thermo Fisher Scientific, Waltham, MA, USA) [56,57,58,59].

After emulsion PCR and purification, the final library was loaded onto an Ion 318 Chip Kit v2 BC (Thermo Fisher Scientific, Waltham, MA, USA) [60,61,62,63,64] and sequenced using a Hi-Q View Sequencing Kit (Thermo Fisher Scientific, Waltham, MA, USA) [65,66,67,68,69] and an Ion Torrent Personal Genome Machine (Thermo Fisher Scientific, Waltham, MA, USA) [70,71,72] at the Next-Generation Sequencing Center of Kyungpook National University (KNU NGS Center).

The Torrent Suite v 5.0 (Thermo Fisher Scientific, Waltham, MA, USA) [73,74] software was used to trim the adaptor sequences. Low-quality reads were removed to obtain a Phred quality score of >30 from the sequenced data. The primer-trimmed files were imported into Quantitative Insights Into Microbial Ecology 2 (QIIME 2) (version 2023.2) [75] in the Casava 1.8 single-end demultiplexed format for further processing using different algorithms (implemented in QIIME 2), which was also run using the 1% distance (99%) reference sequence from the SILVA QIIME release for Eukaryote 2 (version 2022.11.29) [76] to analyze the gut ecoinformation.

Sequencing reads were analyzed using the dada2 plugin in QIIME 2 to generate amplicon sequence variants. A trim length of 350 base pairs was used, and at least one read was required to pass filtering. Amplicon sequence variants with an abundance of <0.1% of the mean sample depth were removed from the analysis.

The output files from QIIME 2 were converted to phyloseq objects using the phyloseq [77] and qiime2r [78] packages in R and then analyzed using ggplot2 [79]. A bar chart was generated using the plot_composition function of the microbiome package with options for 1/100 detection and 10–12/100 prevalence. Alpha diversity was visualized using the plot_richness function of the phyloseq package, and statistical significance was calculated using the ggsignif package [80]. For beta diversity, calculation of the Aitchison and Bray–Curtis distances and redundancy analysis were performed using the microViz package [81], and side box plots were created using the ggside package [82]. Linear discriminant analysis effect size (LEfSe) analysis was performed at the log10 level using the microbiomeMarker package [83].

## 3. Results

### 3.1. Relative Abundance

At the phylum level, the gut of captive1 long-tailed goral predominantly comprised five phyla: Firmicutes (~72.75%), Bacteroidota (~17.71%), Euryarchaeota (~4.20%), Planctomycetota (~2.53%), and Actinobacteriota (~1.96%). The three phyla that dominated the gut of captive2 long-tailed goral were Firmicutes, Bacteroidota, and Euryarchaeota, accounting for ~70.99%, 27.92%, and 1.09%, respectively. The seven phyla that dominated the gut of wild long-tailed goral were Proteobacteria (~38.91%), Actinobacteriota (~23.56%), Bacteroidota (~17.77%), Firmicutes (~13.60%), Acidobacteriota (~1.91%), Euryarchaeota (~1.36%), and Myxococcota (~1.06%).

**Figure 1:**
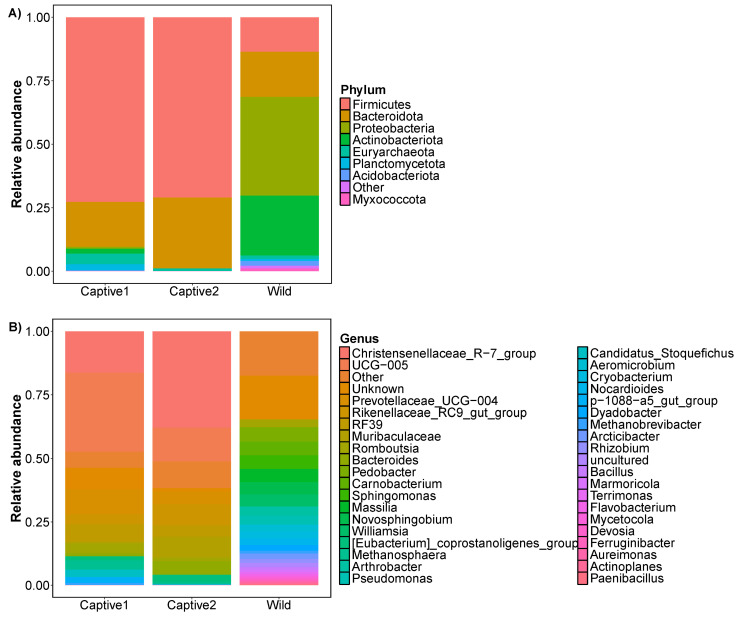
Bar charts showing the relative abundance of total gut 16S rRNA gene sequences classified at the (**A**) phylum and (**B**) genus levels.

At the genus level, the gut of captive1 long-tailed goral predominantly comprised 23 genera: UCG-005 (~31.09%), Christensenellaceae_R-7_group (~16.30%), Prevotellaceae_UCG-004 (~9.86%), RF39 (~6.94%), *Romboutsia* (~3.96%), Rikenellaceae_RC9_gut_group (~3.87%), *Methanosphaera* (~3.39%), Candidatus_*Stoquefichus* (~2.82%), p-1088-a5_gut_group (~2.38%), [*Eubacterium*]_*coprostanoligenes*_group (~1.60%), *Rhodococcus* (~1.53%), *Bacteroides* (~1.20%), *Methanobrevibacter* (~0.81%), *Acetitomaculum* (~0.73%), *Acinetobacter* (~0.68%), Family_XIII_AD3011_group (~0.54%), *Arthrobacter* (~0.42%), *Monoglobus* (~0.25%), *Cerasicoccus* (~0.17%), *Mogibacterium* (~0.15%), *Pirellula* (~0.15%), *Alistipes* (~0.13%), and UCG-010 (~0.08%). The 23 genera that dominated the gut of captive2 long-tailed goral were Christensenellaceae_R-7_group (~37.88%), UCG-005 (~13.32%), Rikenellaceae_RC9_gut_group (~8.25%), *Streptococcus* (~5.63%), *Bacteroides* (~5.37%), Prevotellaceae_UCG-004 (~5.11%), RF39 (~4.50%), [*Eubacterium*]_*coprostanoligenes*_group (~3.02%), *Romboutsia* (~1.51%), *Methanosphaera* (~0.84%), *Alistipes* (~0.74%), *Clostridia*_UCG-014 (~0.66%), *Ruminococcus* (~0.48%), Lachnospiraceae_NK3A20_group (~0.45%), *Acetitomaculum* (~0.42%), *Monoglobus* (~0.41%), *Mogibacterium* (~0.41%), Family_XIII_AD3011_group (~0.31%), *Methanobrevibacter* (~0.26%), UCG-010 (~0.24%), dgA-11_gut_group (~0.18%), *Turicibacter* (~0.16%), and *Izemoplasmatales* (~0.12%). The 23 genera that dominated the gut of wild long-tailed goral were *Pedobacter* (~5.66%), *Carnobacterium* (~5.36%), *Sphingomonas* (~5.35%), *Massilia* (~5.25%), *Novosphingobium* (~4.89%), *Williamsia* (~4.62%), *Pseudomonas* (~3.45%), *Arthrobacter* (~3.43%), *Romboutsia* (~3.08%), *Aeromicrobium* (~2.79%), *Cryobacterium* (~2.67%), *Nocardioides* (~2.66%), *Dyadobacter* (~2.25%), *Arcticibacter* (~2.11%), *Allorhizobium*-*Neorhizobium*-*Pararhizobium*-*Rhizobium* (~1.65%), *Chryseobacterium* (~1.56%), *Methanobrevibacter* (~1.05%), *Acinetobacter* (~1.05%), *Bacillus* (~1.04%), *Marmoricola* (~0.94%), *Terrimonas* (~0.82%), *Flavobacterium* (~0.79%), and *Mycetocola* (~0.73%) (Figure 1 and Table S1).

### 3.2. Alpha Diversity

**Figure 2:**
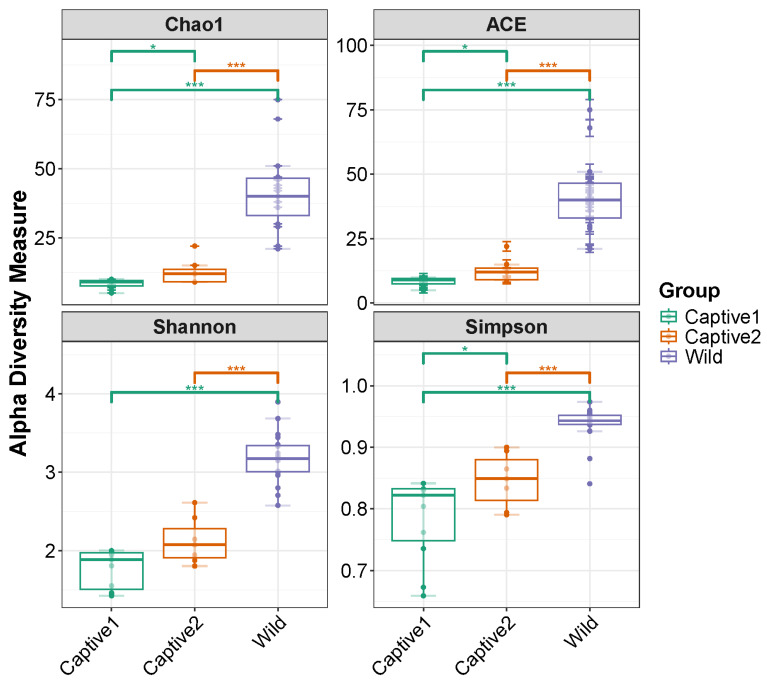
Gut alpha diversity of captive1, captive2, and wild long-tailed gorals. * *p* < 0.05, *** *p* < 0.001.

The gut alpha diversity of captive and wild long-tailed gorals was compared using four indices (Chao1, ACE, Shannon, and Simpson). Except for the Shannon index, all three indices were higher in the captive2 group than in the captive1 group and higher in the wild group than in both captive groups. Except for the Shannon index, all three indices showed a significant difference between the two captive groups *p* < 0.05); however, the difference was at the *** *p* < 0.001 level between the two captive groups and the wild group (Figure 2).

### 3.3. Beta Diversity

**Figure 3:**
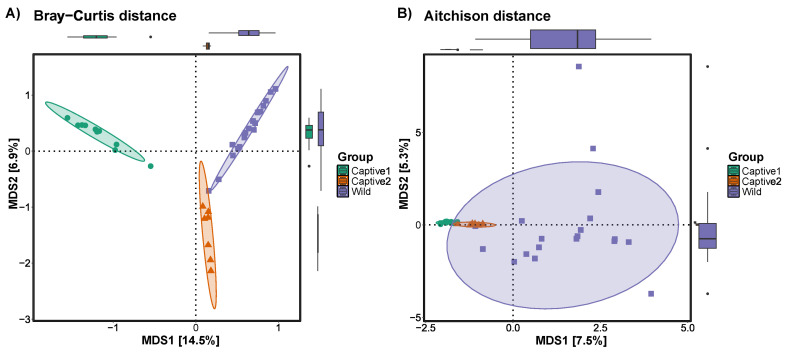
Beta diversity plots were calculated using the (**A**) Bray–Curtis and (**B**) Aitchison distances to compare captive and wild long-tailed gorals.

Beta diversity was measured by a multidimensional scaling method using Bray–Curtis and Aitchison distances. In the plot calculated using the Bray–Curtis distance, clear differences were observed among the captive1, captive2, and wild groups, with captive2 being closer to the wild group than captive1. In the plot with Aitchison distance, the captive1 and 2 groups appeared similar and clustered densely, whereas the wild group showed wide clustering (Figure 3).

### 3.4. LEfSe

**Figure 4:**
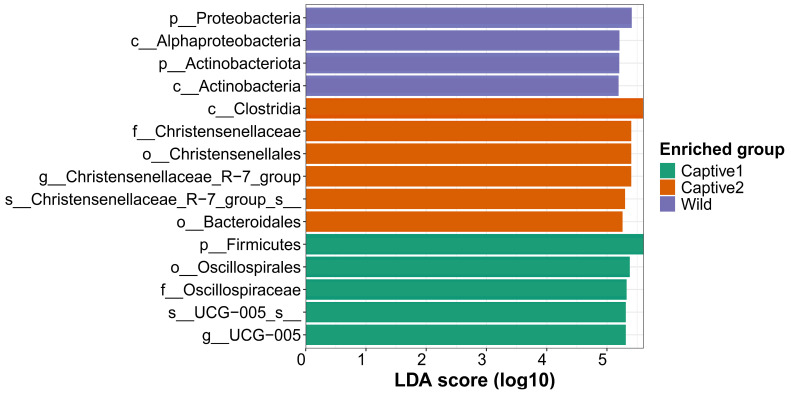
Linear discriminant analysis effect size on a log10 scale illustrating differences in LDA scores between captive and wild long-tailed gorals.

LEfSe was representative of taxa in the captive1, captive2, and wild groups. The genus UCG-005, family Oscillospiraceae, and phylum Firmicutes were most represented in the captive1 group; the order Bacteroidales and the genus Christensenellaceae_R-7_group, family Chrisensenellaceae, and order Christensenellales were most represented in the captive2 group; and the classes Alphaproteobacteria and Actinobacteria were most represented in the wild group (Figure 4).

### 3.5. Redundancy Analysis

**Figure 5:**
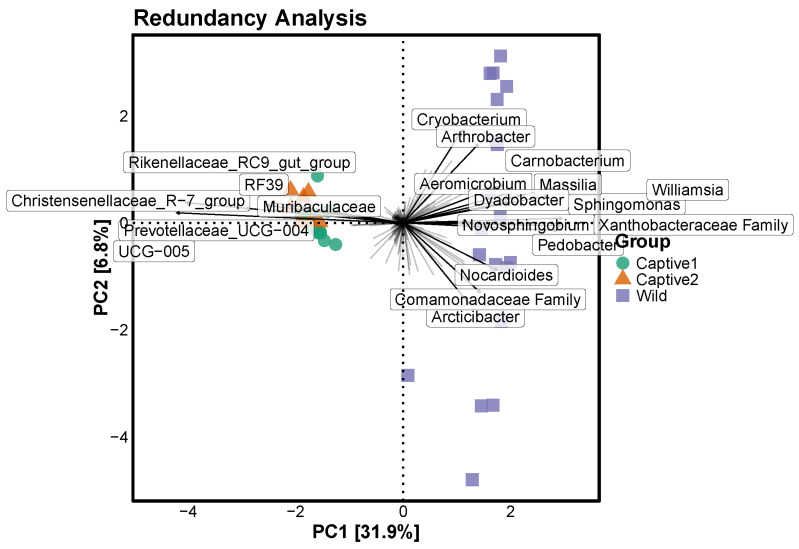
RDA illustrating differences in abundance between captive and wild long-tailed gorals.

The redundancy analysis was limited to the top 20 bacterial genera and revealed the dominant bacterial genera in each group. The captive and wild groups were divided into opposite directions. The two captive groups were densely clustered, whereas the wild group was widely distributed. The representative taxa of the two captive groups were Muribaculaceae, Rikenellaceae_RC9_gut_group, Christensenellaceae_R−7_group, Prevotellaceae_UCG−004, and UCG-005. The representative taxa of the wild group were *Cryobacterium*, *Arthrobacter*, *Carnobacterium*, *Aeromicrobium*, *Massilia*, *Dyadobacter*, *Williamsia*, *Sphingomonas*, *Novosphingobium*, family Xanthobacteraceae, *Pedobacter*, *Nocardioides*, family Comamonadaceae, and *Arcticibacter* (Figure 5).

## 4. Discussion

This study aimed to contribute to the conservation of long-tailed gorals using systematic analysis of their gut microbiomes. In long-tailed gorals, the change in microecology (change in gut microorganisms) resulting from a change in macroecology (living in the wild to living in captivity) led to the loss of >60% of bacterial phyla in the gut. The wild group initially contained 17 bacterial phyla. However, in the captive1 group, only seven phyla remained, and the captive2 group had three phyla. This is a large change and is the reason why we must be cautious when raising long-tailed gorals in captivity. Increased vigilance is needed for long-tailed goral events, in which hundreds to thousands of sudden deaths have been recorded in Korea. Ex situ conservation of long-tailed gorals aims to reintroduce them into the wild. However, massive changes in microecology make our goal of ex situ conservation seem distant. Therefore, we must evaluate the reasons for these changes.

The bacterial phyla Proteobacteria and Actinobacteria are commonly found in animal guts [84]. Proteobacteria have a reputation for causing disease or imbalance in the gut [85]; however, because research on pathogens far outweighs research on the gut microbiome, they may play a useful role in the animal gut [86]. Proteobacteria include some pathogens, but in the natural environment, most play a role in the decomposition of compounds [87]. The long-tailed goral consumes various plant species in the wild and must break down plant-based compounds. Long-tailed gorals sometimes lick dirt and rocks, but this does not explain >60% of their gut microbiome. It is reasonable to assume that the biggest change from wild to captive-bred food sources is the change in the gut microbiome during the digestion of this food source. The abundance of Proteobacteria may have decreased because there is no reason to break down various compounds by moving from plant food sources to limited food sources. Generally, Actinobacteria produce antibiotics and inhibit the growth of other microorganisms [88]. We hypothesized that long-tailed gorals produce antibiotics that participate in the immune system. The relative abundance results were statistically supported by LEfSe on a log10 scale.

At the bacterial genus level, the dominance of certain genera was high in captive groups, but each genus was distributed evenly in the wild group. This could provide evidence that wild long-tailed gorals consume various types of food. Because they move around constantly and consume different food sources, each bacterial genus can become equalized. In captive long-tailed gorals that consumed the same diet, the abundance of specific bacterial genera increased, and evenness across all bacterial genera decreased. This result indicates that to maintain the wild nature of long-tailed gorals, each bacterial genus must be maintained as evenly as possible.

We found that not only the composition but also the diversity of the gut microbiome changed with changes in the food source. Higher diversity of the gut microbiome is believed to correlate with a healthier gut [89]. In long-tailed gorals, the diversity of the gut microbiome of the captive-bred groups decreased; therefore, their health must have deteriorated. However, they appear healthy and live healthy lives. Although captive2 was fed a more diverse diet than captive1 and was expected to be healthier, no differences in appearance were observed between the two breeding groups. However, the diversity of the gut microbiome that long-tailed gorals originally had in the wild decreased when they were raised in captivity. Because of this decrease in diversity, the gut microbiome of the long-tailed goral released from ex situ conservation, reintroduced into the wild, and fed on wild leaves needs time to return to its original high diversity state. Recent studies have shown a close relationship between the gut microbiome and the brain [90], and rapid changes in the gut microbiome may affect the brain. To reduce this effect, it is necessary to provide various types of food to long-tailed gorals and shorten their adaptation time.

The beta diversity results indicated the need for various types of food. When plotting the Bray–Curtis distance, captive2 showed several individuals located close to the long-tailed goral of the wild group. Although some individuals were distant from the wild group, in the case of captive1, no individual appeared adjacent to the long-tailed goral of the wild group. Similarly, when plotting with the Aitchison distance, the captive2 group appeared adjacent to the wild group. In the wild, long-tailed gorals spread out and ate various types of food, indicating an incredibly diverse gut microbiome.

RDA divided wild long-tailed gorals into three main groups. In group 1, the genera *Cryobacterium* and *Arthrobacter* were mainly distributed in the gut. *Cryobacterium* is a recently discovered taxon that was isolated from glaciers and produces enzymes that adapt to cold [91,92,93,94,95]. *Arthrobacter* also produces cold-adapted enzymes [96,97,98,99,100]. Therefore, we hypothesized that the long-tailed goral group 1, which contains *Cryobacterium* and *Arthrobacter*, lives in colder places than other groups and is adapted to the cold. Furthermore, the group lives and forms their home range mainly in alpine areas where temperatures are relatively low. In group 2, the genera *Norcardioides* and *Arcticibacter*, and family Comamonadaceae were mainly distributed in the gut. *Arcticibacter* is not a well-known bacterial genus, but some strains of *Norcardioides* have been isolated from hot springs [101], and many strains of Comamonadaceae have been isolated from hot springs [102,103,104,105]. In contrast to group 1, group 2 contained heat-resistant strains in the gut. We hypothesized that group 2 adapted to and lived in areas with higher temperatures and lower elevations than group 1. In group 3, *Carnobacterium*, *Aeromicrobium*, *Massilia*, *Dyadobacter*, *Williamsia*, *Sphingomonas*, *Novospingobium*, and *Pedobacter* were mainly distributed in the gut. Thus, long-tailed gorals live in groups divided by height, such as highlands, midlands, and lowlands. On the basis of these results, it appears that the distribution of plants according to altitude increased the diversity of the gut microbiome of long-tailed gorals. There appears to be a close relationship between plant succession and long-tailed goral. These results allow us to examine the changes that occur when long-tailed gorals, which used to live at different heights and come in contact with various plant types, consumed a unified height and a unified diet during ex situ conservation. To prepare for the extinction crisis, (1) the breeding areas of long-tailed gorals should be divided into highlands, midlands, and lowlands and (2) more organizations should be included in ex situ conservation. Species extinction cannot be prevented if national organizations do not act. National organizations should take the lead in nurturing organizations that prevent extinction.

The need to increase the long-tailed goral at more sites and institutions arises not only from various weather conditions but also from sudden abnormal climate events. Typically, long-tailed gorals live in large mountain ranges called Baekdudaegan. The Baekdudaegan Mountain Range experiences the Foehn phenomenon, which results in different environments on the east and west sides of the mountain range. Natural disasters, such as wildfires, occur because of high temperatures and dry winds caused by the Foehn phenomenon, which burn down the habitats of long-tailed gorals. Another natural disaster is heavy snow caused by abnormal weather. The large-scale mortality of long-tailed gorals has been occurring since the 1960s, and it is sometimes related to heavy snowfall. In a conservation area in Uljin, snow and rain due to the Foehn phenomenon formed an ice layer at extremely low temperatures. Movement in the snow-covered area became impossible because the long-tailed goral had short legs and could not feed. In Korea, the large-scale deaths of animals due to these natural disasters has not yet been studied in detail. However, in Mongolia, these natural disasters are called “dzud”, and the deaths of livestock due to extremely low temperatures and heavy snow are treated seriously [106,107]. In Korea, we must treat these natural disasters seriously and prepare for them. The Ministry of Environment of Korea recorded 700–900 long-tailed gorals. When the military border areas between South Korea and North Korea are combined, the number can be estimated at ~2000 at most. Last winter, ~1022 long-tailed gorals died because of heavy snow caused by abnormal weather; therefore, if the records are accurate, long-tailed gorals are close to extinction. To prevent their extinction, we propose that long-tailed gorals should be conserved ex situ in more diverse environments, locations, and institutions. Their extinction can be prevented if large numbers are reintroduced into nature. Government-level support is needed from many organizations for the ex situ conservation of long-tailed gorals. At the government level, we should pay more attention to endangered species and provide more support.

After direct state support is provided, indirect care for the long-tailed goral will also need to be provided. Forest roads have been created in most mountains in Korea, causing confusion for long-tailed gorals as they move around. In addition, hiking trails are overdeveloped and long-tailed gorals are severely affected by disturbance due to people collecting forest products and hunting. Additionally, the movement paths of long-tailed gorals are restricted due to the barriers installed by farmers to prevent the intrusion of wild animals, and they sometimes die after becoming entangled in the net barriers. In order to conserve long-tailed gorals, these disturbances must be reduced as much as possible, and conservation areas and off-limits areas must be established and protected to prevent their complete extinction. Representative examples include national parks and conservation areas. A national park where many people can monitor long-tailed goral disturbances and manage their habitat could be very helpful to the long-tailed goral’s survival. Many long-tailed goral habitats in Korea have been designated as national parks, but there are areas that have not yet been designated, so we propose to conduct additional research on those areas and designate them as national parks as soon as possible. Another proposal is to systematically reintroduce long-tailed the goral. It will also be necessary to select various locations each year and determine the number of long-tailed gorals to be reintroduced so that they can adapt and live in a variety of environments. As the number of long-tailed gorals in Korea has decreased due to the overwhelming number of deaths, the number that has died will need to be restored directly or indirectly. We propose to reintroduce an appropriate number of male and female long-tailed mountain goats each year to each protected area designated as a national park.

Our findings indicate that changes in the gut microbiome of long-tailed gorals must be tracked when ex situ conservation is performed. Additional research should be conducted to minimize changes in the gut microbiome of wild long-tailed gorals when they are raised by humans. Currently, we only feed wild long-tailed gorals, raise them, and check their external appearance. They need to be managed more systematically and scientifically. Systematic management of gut microbiomes in the wild, in captivity, and at breeding locations is urgently needed. The systematic management we propose is not limited to the bacterial microbiome. The fungal microbiome can be used for management as well, and it would be even better if research was conducted on both the bacterial and fungal microbiome. Our study was conducted targeting the V4–V5 region of the bacterial 16S rRNA region, but it would be sufficiently manageable to conduct research targeting other regions as well. If possible, it would be a good idea to conduct research by amplifying the full length of the 16S rRNA region.

## 5. Conclusions

We compared the bacterial microbiomes of wild long-tailed gorals and two types of long-tailed gorals that were bred in captivity. The gut microbiomes of long-tailed gorals that consumed a more diverse diet were more diverse. Analysis of the gut microbiomes revealed that long-tailed gorals are distributed in relatively high, middle, and relatively low areas of the mountain. For the first time, it was revealed that long-tailed goral goats are divided into three groups depending on their height, and that the bacterial intestinal microbial community changes significantly when long-tailed gorals are raised through ex situ conservation. Our research has shown that in order to conserve long-tailed gorals closer to the wild, we need to understand the flora that varies depending on mountain height and provide a variety of plants as food sources for long-tailed gorals.

We suggest that the gut microbiome provides more information about the ecology of long-tailed gorals and plays an important role in their conservation.

## Figures and Tables

**Table 1:** Information about fecal samples collected by region.

Region	No. of Samples	Type of Samples
Seoraksan National Park	19	Wild
Northern Conservation Center	11	Captive1
Long-tailed Goral & Musk Deer Center	7	Captive2
Total	37	

## Data Availability

The microbiota data used in this study are available on figshare: https://doi.org/10.6084/m9.figshare.25910995 (accessed on 28 May 2024) and BioProject PRJNA1133272 on NCBI.

## Supplementary Materials

The following supporting information can be downloaded at: https://www.mdpi.com/article/10.3390/microorganisms12071419/s1, Table S1: ASV table.
